# The level of human epididymis protein 4 is associated with poor prognosis in patients with ejection fraction preserved heart failure

**DOI:** 10.3389/fcvm.2025.1530271

**Published:** 2025-08-18

**Authors:** Yi Tang, Yu Chen, Xiao Jiao Huang, Zheng Qi Hu, Yon Jun Hu, Si-ling Peng, Hong-wei Pan

**Affiliations:** ^1^ Department of Cardiology, Hunan Provincial People’s Hospital, The First Affiliated Hospital of Hunan Normal University, Clinical Medicine Research Center of Heart Failure of Hunan Province, Hunan Normal University, Changsha, China; ^2^ Department of Cardiology, The First Affiliated Hospital of Hunan Normal University Hunan Provincial People's Hospital, Hunan Normal University, Changsha, China

**Keywords:** ejection fraction preserved heart failure, human epididymis protein 4, prognosis, biomarker, fibrosis

## Abstract

**Introduction:**

The prognostic value of human epididymis protein 4 (HE4) in patients with heart failure with preserved ejection fraction (HFpEF) is unknown.

**Methods:**

Patients diagnosed with HFpEF in the Department of Cardiovascular Medicine of Hunan Provincial People's Hospital from January 2021 to August 2022 were prospectively enrolled in the study. Serum levels of HE4 were measured using a chemiluminescence microparticle immunoassay. Endpoint events included readmission for heart failure and cardiovascular death.

**Results:**

A total of 170 patients with HFpEF were included in the study; among them, 86 (50.6%) were male, with a mean age of 68.9 ± 0.8 years. A total of 71 patients experienced endpoint events, including 67 patients with rehospitalizations for heart failure and 4 patients with cardiovascular deaths, with an average follow-up of 18.0 ± 0.8 months. Multivariate Cox regression analysis revealed that HE4 was an independent predictor of endpoint events (HR = 1.009, 95% CI 1.002–1.015, *P* = 0.010). The Kaplan–Meier survival curves revealed that the risk of endpoint events was significantly higher in patients with HE4 > 42.45 pmol/L than in those with HE4 ≤ 42.45 pmol/L (HR = 2.66, 95% CI 1.37–5.17, *P* < 0.01). After adjusting for age and gender, the HR was 2.48 (95% CI 1.21–5.08, *P* < 0.05).

**Conclusion:**

HE4 is an independent predictor of heart failure rehospitalization and cardiovascular death in patients with HFpEF.

## Introduction

Heart failure (HF) represents the end stage of various cardiovascular diseases. It is a syndrome characterized by insufficient perfusion of organs and tissues due to various structural and functional cardiac disorders. Myocardial damage and excessive cardiac workload are the primary causes. HF is classified into three categories based on the left ventricular ejection fraction (LVEF): heart failure with reduced ejection fraction (HFrEF) (LVEF ≤ 40%), heart failure with mildly reduced ejection fraction (HFmrEF) (LVEF 41%–49%), and heart failure with preserved ejection fraction (LVEF >= 50%) (1). Studies have shown that 56.5% of outpatients with HF have HFpEF (2). The prognosis for HFpEF patients is poor, with no significant difference compared to HFrEF patients, and the 5-year survival rate after a first episode is 43% (3–5). Therefore, evaluating the prognosis of HFpEF patients is important.

Biomarkers play an important role in the prognostic assessment of HF. NT-proBNP is widely used in the prognostic assessment of HF. However, NT-proBNP levels are usually lower in patients with HFpEF than in patients with HFrEF (6) or are even partially within the normal range (7). Moreover, NT-proBNP levels are susceptible to various factors and diseases such as renal function, age, atrial fibrillation, and pulmonary embolism, which can reduce its prognostic value (8).

HE4, also known as whey acidic protein four-disulfide core domain protein 2, was first discovered in epididymal tissue. It is involved in sperm maturation (9). Previously, HE4 was often used in clinical diagnosis of gynecologic malignancies (10). In 2013, de Boer et al. reported that HE4, a novel marker of HF, was correlated with the prognosis and severity of acute heart failure (11). In 2017, Piek A et al. reported that the serum HE4 could predict the prognosis of patients with chronic heart failure (12). Our previous findings revealed that HE4 can predict the prognosis in patients with ischemic cardiomyopathy (13). All of these studies showed that serum HE4 was associated with prognosis in patients with HFrEF, possibly because HE4 is involved in myocardial fibrosis. Myocardial fibrosis also plays a significant role in HFpEF (14). However, whether serum HE4 levels can predict the prognosis of HFpEF patients is unknown. The present study aimed to investigate the predictive value of HE4 for the prognosis of patients with HFpEF.

## Methods

### Study population

This study prospectively enrolled 178 patients with HFpEF who were hospitalized in the Department of Cardiovascular Medicine of Hunan Provincial People's Hospital from January 2021 to August 2022. HFpEF was diagnosed according to the following criteria (1): (1) HF symptoms and/or signs; (2) LVEF ≥50%; (3) objective evidence of cardiac structural and/or functional abnormalities consistent with LV diastolic dysfunction/elevated LV filling pressures (including elevated natriuretic peptide). Patients with the following conditions were excluded: (1) patients with known malignancy; (2) end-stage renal disease (patients requiring dialysis treatment or with eGFR <30 ml/min/1.73 m^2^); (3) severe hepatic insufficiency [Child‒Pugh class B, or C]; (4) women preparing for or during pregnancy; (5) patients with a life expectancy of less than 1 year for noncardiac reasons.

### Clinical assessment and follow-up

Baseline clinical data such as gender, age, biochemical tests, drug assessments and echocardiography were collected within two days after the patient was admitted to the hospital. NT-proBNP levels were measured by chemiluminescent immunoassay (Vantec Carey, Xiamen, China) at the Department of Laboratory Medicine, Hunan Provincial People's Hospital. Patients were followed up at 1, 3, 6, 12, and 18 months after discharge from the hospital, and the occurrence of endpoint events at the follow-up time was recorded. Endpoint events were defined as rehospitalization for HF and cardiovascular death. The follow-up cut-off was October 1, 2023.

### HE4 measurement

Peripheral venous blood samples were collected the following morning after admission. The blood samples were centrifuged at 1,000 rpm for 15 min, after which the upper serum was collected and frozen at −80°C. Serum HE4 levels were determined by chemiluminescent immunoassay (Abbott, Germany) at the Department of Nuclear Medicine, Hunan Provincial People's Hospital. The HE4 assay is a two-step immunoassay for the quantitative determination of the HE4 antigen in human serum using chemiluminescent microparticle immunoassay technology with flexible assay protocols. The detectable dose range of human HE4 was 20–1,500 pmol/L. The intra and inter-assay coefficients of variation were less than 3.5 and 4.9%, respectively.

### Statistical analysis

SPSS 19.0 statistical software was used for analysis. Continuous variables with a normal distribution were expressed as the mean ± standard deviation, and continuous variables with a nonnormal distribution are represented by the median and quartile (IQR). Independent Student's *t*-test were used for comparisons between groups of normally distributed measurements, and the Mann‒Whitney-*U* test was used for comparisons between groups of non-normally distributed measurements. Categorical variables and ordinal variables were expressed as frequencies (percentages), and group comparisons for categorical variables were performed using the chi-square test. Bivariate correlation analysis was performed using Pearson or Spearman correlation coefficients. Receiver Operating Characteristic (ROC) Curve was used to judge the performance of the variables in prognostic prediction. Univariate and multivariate Cox proportional hazards models and Kaplan‒Meier curve were used for survival analysis. A two-sided *P* < 0.05 was defined as a statistically significant difference.

## Results

### Baseline data and clinical characteristics of patients

**Figure 1:**
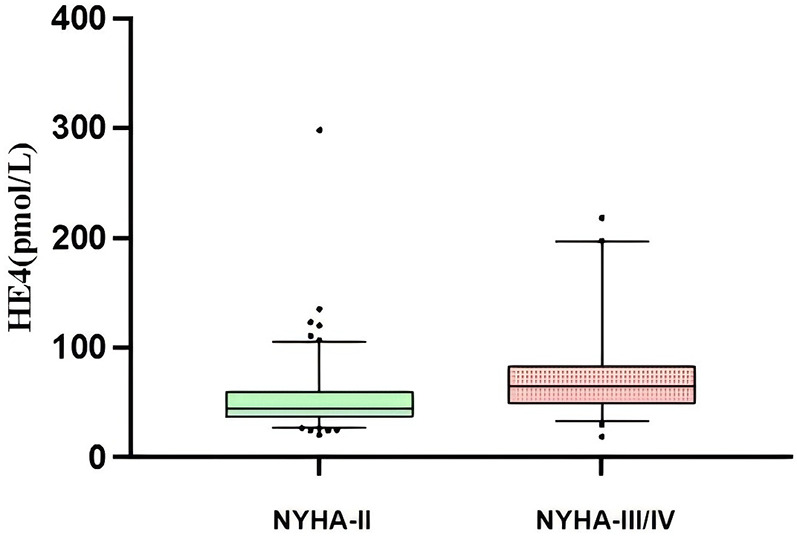
Sertun HE4 levels in HFpEF patients with different NYHA-FC.

A total of 178 patients with HFpEF were included in this study, 8 patients were not included in the study due to loss of follow-up, ultimately 170 patients were included in the analysis, 109 patients had coronary heart disease, 7 patients had hypertensive heart disease, 6 patients had hypertrophic cardiomyopathy, and 48 patients had multiple aetiologies (combining at least two aetiologies and more). Among them, 86 (50.6%) were male, and the mean age was 68.9 ± 0.8 years. A total of 71 patients experienced endpoint events, including 67 patients with rehospitalizations for heart failure and 4 patients with cardiovascular deaths, with a mean follow-up time of 18.0 ± 0.8 months. The differences between the two groups were not statistically significant (all *P* > 0.05) in terms of gender, age, body mass index (BMI), New York Heart Association functional classification (NYHA-FC), LVEF, left atrial volume index, medication history, and baseline medical history (hypertension, diabetes mellitus, chronic obstructive pulmonary disease, and dyslipidemia). Compared with the event group, the non-event group had lower serum HE4 levels and higher eGFR levels (*P* < 0.01) (Table 1). Furthermore, as NYHA-FC increased, HE4 levels became significantly higher (NYHA-FC II 52.0 ± 2.8 pmol/L vs. NYHA-FC III/IV 71.9 ± 5.7 pmol/L, *P* < 0.001) (Figure 1).

**Table 1:** Baseline characteristics of patients with HFpEF with and without events.

Variables	No events group	Events group	Total	*P* value
	(*n* = 99)	(*n* = 71)	(*n* = 170)	
Age, (y)	67.9 ± 1.1	70.4 ± 1.1	68.9 ± 0.8	0.096
Gender, *n* (%)	49 (57%)	37 (43%)	86 (50.6%)	0.736
BMI, (Kg/m^2^)	24.3 ± 0.4	23.5 ± 0.4	23.9 ± 0.3	0.327
Systolic blood pressure, (mmHg)	129.8 ± 1.7	128.1 ± 1.9	129.1 ± 1.2	0.625
Smoking, *n* (%)	24 (48%)	26 (52%)	50 (29.4%)	0.081
Hypertension, *n* (%)	73 (58.4%)	52 (41.6%)	125 (73.5%)	0.942
Diabetes mellitus, *n* (%)	36 (53.7%)	31 (46.3%)	67 (39.4%)	0.337
Dyslipidaemia, *n* (%)	41 (57.7%)	30 (42.3%)	71 (41.8%)	0.913
COPD, *n* (%)	7 (43.8%)	9 (56.3%)	16 (9.4%)	0.217
NYHA-FC				0.230
II, *n* (%)	75 (62%)	46 (38%)	121 (71.2%)	
III, *n* (%)	21 (51.2%)	20 (48.8%)	41 (24.1%)	
IV, *n* (%)	3 (37.5%)	5 (62.5%)	8 (4.7%)	
WBC, (10^9^/L)	6.8 ± 0.2	7.1 ± 0.3	6.9 ± 0.2	0.486
ALT, (U/L)	31.8 ± 5.9	20.6 ± 1.7	27.1 ± 3.5	0.035
eGFR, (ml/min/1.73 m^2^)	88.2 ± 1.4	81.5 ± 1.7	85.4 ± 1.1	0.001
LDL-C, (mmol/L)	2.0 ± 0.1	2.1 ± 0.1	2.0 ± 0.1	0.759
CK-MB, (U/L)	17.6 ± 2.5	21.3 ± 4.1	19.2 ± 2.3	0.655
HE4, (pmol/L)	51.4 ± 2.4	67.1 ± 5.3	58.0 ± 2.7	0.002
NT-proBNP, (ng/L)	1,301.6 ± 136.0	1,425.6 ± 294.7	1,353.7 ± 146.4	0.659
E/e′	12.5 ± 0.4	13.9 ± 0.6	13.1 ± 0.4	0.094
LAVI, (ml/m^2^)	37.3 (30.8–45.2)	38.6 (31.7–43.8)	37.7 (31.2–55.0)	0.956
LVEF, (%)	62.2 ± 0.7	61.1 ± 0.9	61.7 ± 0.6	0.304
β-Blocker, *n* (%)	82 (56.6%)	63 (43.4%)	145 (85.3%)	0.284
ACEIs/ARBs/ARNI, *n* (%)	82 (60.3%)	54 (39.7%)	136 (80%)	0.276
SGLT2 inhibitors	17 (17.2%)	15 (21.1%)	32 (18.8%)	0.515
Spirolactone	17 (17.2%)	10 (14.1%)	27 (15.9%)	0.587

The data is presented as *n* (%) for categorical variables, mean (standard deviation) for continuous variables, or median (interquartile range, IQR) for skewed distributions.

ACEI, angiotensin-converting enzyme inhibitor; ALT, alanine aminotransferase; ARB, angiotensin receptor blocker; ARNI, angiotensin receptor and enkephalinase inhibitor; BMI, body mass index; CK-MB, creatine kinase-MB; COPD, chronic obstructive pulmonary disease; E/e′, the ratio of peak anterior flow velocity in the early mitral orifice to e′ mean; eGFR, estimated glomerular filtration rate; HE4 human epididymis protein 4; LDL-C, low-density lipoprotein cholesterol; LAVI, left atrial volume index; LVEF, left ventricular ejection fraction; NT-proBNP N-terminal prohormone of B-type natriuretic peptide; NYHA-FC New York heart association functional classification; SGLT2, sodium-glucose cotransporter 2; WBC, white blood cells.

### Correlation analysis

HE4 levels were positively correlated with age (r = 0.279, *P* < 0.01), NT-proBNP (*r* = 0.465, *P* < 0.01), and WBC (*r* = 0.193, *P* = 0.012), and negatively correlated with eGFR levels (*r* = −0.398, *P* < 0.01) (Table 2).

**Table 2:** Correlation analysis.

Variables	r-value	*P*-value
Age	0.279	0.001
NT-proBNP	0.465	0.001
eGFR	−0.398	0.001
WBC	0.193	0.012

eGFR, estimated glomerular filtration rate; NT-proBNP, N-terminal prohormone of B-type natriuretic peptide; WBC, white blood cells.

### Univariate and multivariate Cox analyses

The results of the univariate analysis revealed that HE4, NYHA-FC, and the eGFR could be used as prognostic indicators for patients with HFpEF (*P* < 0.05). Variables with *P* < 0.05 in the univariate Cox regression analysis (HE4, NYHA-FC, eGFR) and variables that are clinically associated with the prognosis of patients with HFpEF (e.g., age, diabetes, E/e′, and NT-proBNP) were included in the multivariate Cox regression analysis. The method of input variables was used, with the results revealing that the serum HE4 level (HR = 1.009, 95% CI 1.002–1.015, *P* = 0.010) was an independent predictor of endpoint events in patients with HFpEF (Table 3).

**Table 3:** Univariate and multivariate cox regression analyses.

Variables	Univariate analysis			Multivariate analysis		
	HR	95% CI	*P* value	HR	95% CI	*P* value
HE4	1.010	1.005–1.015	<0.001	1.009	1.002–1.015	0.010
Log_10_ NT-proBNP	1.121	0.586–2.145	0.730	0.783	0.385–1.591	0.498
eGFR	0.975	0.958–0.992	0.005	0.986	0.967–1.006	0.175
Age	1.019	0.995–1.044	0.118	1.000	0.973–1.028	0.999
NYHA-FC	1.480	1.009–2.169	0.045	1.324	0.867–2.024	0.194
E/e'	1.034	0.992–1.077	0.111	1.028	0.984–1.074	0.212
Diabetes	1.169	0.730–1.870	0.515	0.881	0.543–1.429	0.607

CI, confidence interval; E/e′, the ratio of peak anterior flow velocity in the early mitral orifice to e′ mean; eGFR, estimated glomerular filtration rate; HE4, human epididymis protein 4; HR, risk ratio; Log10(NT-proBNP), The logarithm of the N-terminal prohormone of B-type natriuretic peptide value based on 10; NYHA-FC, New York heart association functional classification.

### ROC curve analysis

**Figure 2:**
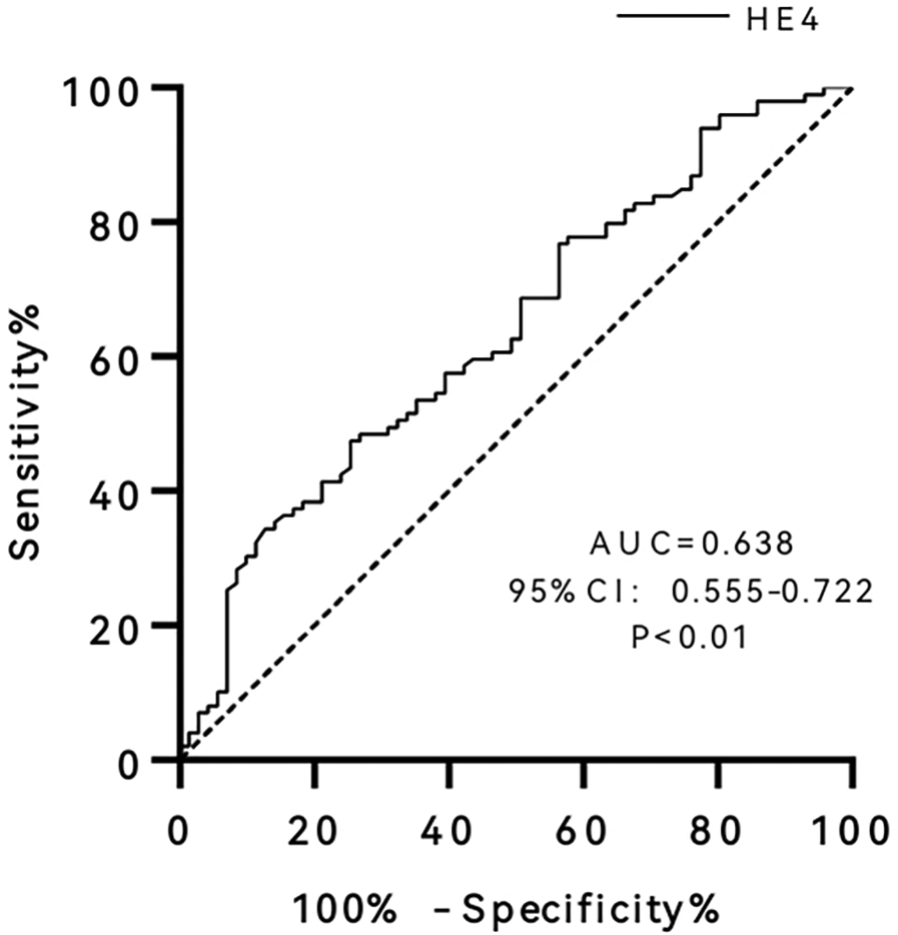
Receiver operating characteristic (ROC) curve of human epididymis protein 4 (HE4). AUC, area under curve; CI, confidence interval.

ROC curves were used to analyze the performance of serum HE4 levels in predicting endpoint events (Figure 2). The results revealed that the AUC of HE4 was 0.638 (95% CI 0.555–0.722, *P* < 0.01), and the optimal cut-off was 42.45 pmol/L, with sensitivity and specificity of 74.6% and 47.5%, respectively.

### Kaplan–Meier survival analysis

**Figure 3:**
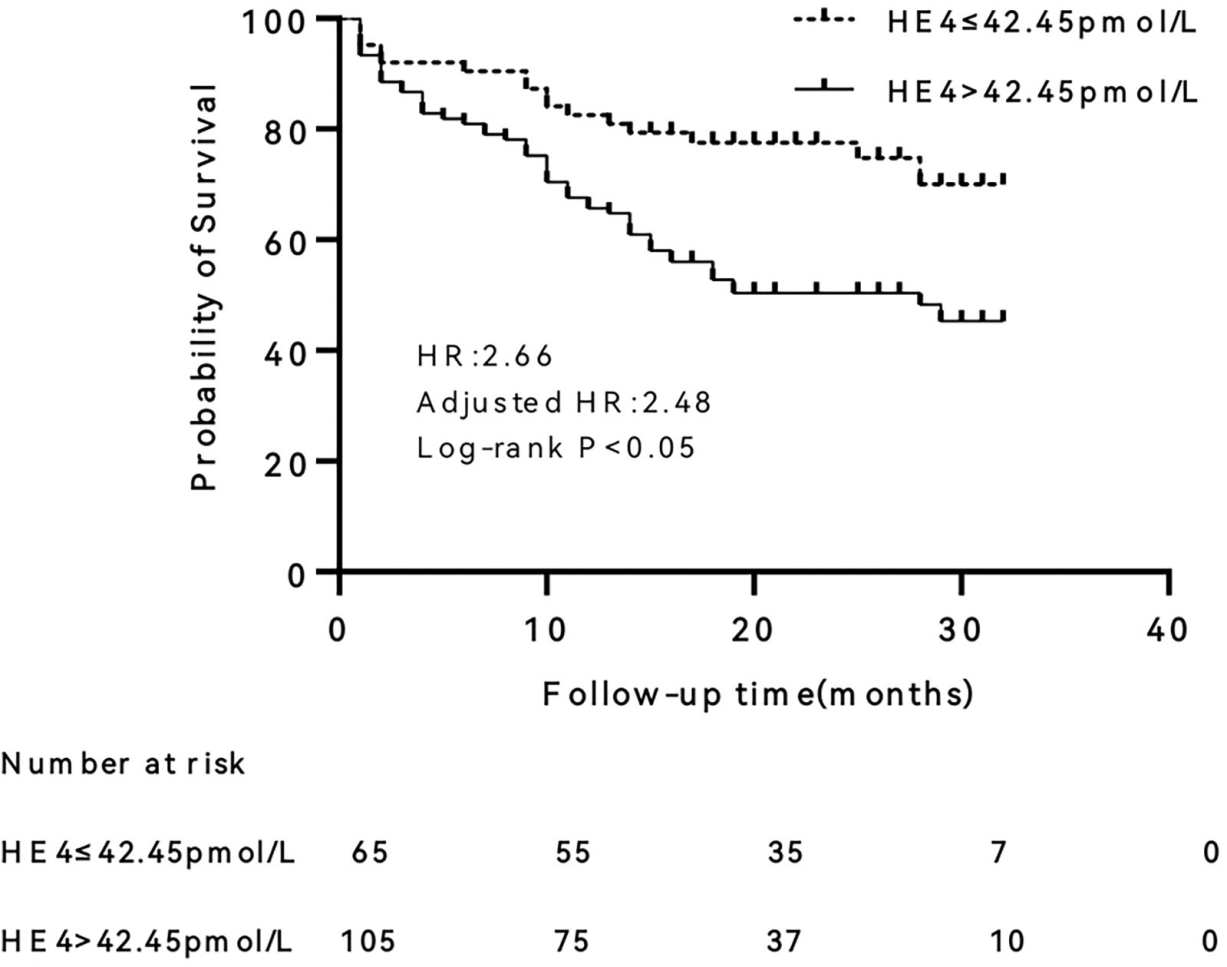
Kaplan–Meier survival curve of human epididymis protein 4 (HE4). HR, hazard ratio.

The differences in survival between the two groups were compared after patients were grouped according to the optimal cut-off point of the ROC curve (Figure 3). The results revealed that patients with HE4 > 42.45 pmol/L had a significantly higher risk of endpoint events than those with HE4 ≤ 42.45 pmol/L (HR = 2.66, 95% CI 1.37–5.17, *P* < 0.01). After adjusting for age and gender, the HR was 2.48 (95% CI 1.21–5.08, *P* < 0.05).

## Discussion

To the best of our knowledge, this is the first study to investigate the predictive value of HE4 for the prognosis of patients with HFpEF. The higher the NYHA-FC, the higher the serum HE4 level was patients with HFpEF. Serum HE4 levels are positively correlated with age and NT-proBNP levels and negatively correlated with eGFR levels. HE4 is an independent predictor of cardiovascular death and heart failure rehospitalization in patients with HFpEF. Patients with serum HE4 levels > 42.45 pmol/L had a 2.48-fold higher risk of endpoint events than patients with serum HE4 levels ≤ 42.45 pmol/L.

HE4 has antiprotease activity and can inhibit a variety of proteases (15). Serum HE4 levels are closely related to the severity and prognosis of patients with HF and are significantly and positively correlated with the levels of Galectin-3, a marker of cardiac fibrosis (11, 12). Yamamoto M et al. reported that HE4 was indirectly involved in cardiac fibrosis by activating cardiac fibroblasts (16), LeBleu et al. reported that HE4 could be involved in renal fibrosis by inhibiting the activities of serine proteases and matrix metalloproteinases and is correlated with the severity of renal fibrosis (17). HFpEF involves complex pathological mechanisms, among which myocardial fibrosis plays a crucial role in the development and progression of the condition (18). In our study, we found that serum HE4 levels increased with the deterioration of cardiac function and were positively correlated with NT-proBNP levels in patients with HFpEF. Therefore, we hypothesized that HE4 may be involved in the development of HFpEF by participating in fibrosis. However, there is no direct evidence of HE4 involved in myocardial fibrosis. Future clinical and basic studies need to be conducted to demonstrate the direct evidence of this mechanism.

Some previous studies reported that BNP or NT-proBNP levels were associated with poor prognosis in patients with HFpEF (19, 20). However, other studies have shown that NT-proBNP levels have no predictive value for the prognosis of HFpEF patients (21, 22). Our study also revealed that NT-proBNP is not an independent predictor of endpoint events in patients with HFpEF, and there is no statistically significant difference in NT-proBNP levels between the event and non-event groups. More importantly, our study found that HE4 is an independent predictor of heart failure rehospitalization and cardiovascular death in patients with HFpEF. Of course, we should be aware of the limitations of the clinical significance of HE4. The hazard ratio of HE4 is 1.009, which means only a 0.9% increase in risk per unit increase in HE4. In addition, the sensitivity and specificity of HE4 were 74.6% and 47.5%, respectively, when the optimal cut-off was 42.45 pmol/L. This low specificity would potentially result in misclassification of many patients as high-risk.

Our study has several limitations. First, HE4 levels were measured only at baseline, and it is unclear whether changes in HE4 levels after treatment can predict the prognosis of patients with HFpEF. Second, the sample size of this study was relatively small, and the follow-up time was relatively short, which results in inadequate statistical power. Our research is a single-center study, and the results may not be applicable to other hospitals or regions. A multicenter, large-scale sample is needed to further confirm that HE4 is an independent predictor of poor prognosis in patients with HFpEF.

## Conclusion

HE4 is an independent predictor of heart failure rehospitalization and cardiovascular death in patients with HFpEF.

## Data Availability

The dataset used in this study has restrictions related to patient privacy and data confidentiality, as it contains sensitive medical information. Access to the dataset is limited to authorized researchers and requires institutional approval, ensuring compliance with ethical guidelines and regulations for handling patient data. Requests to access the datasets should be directed to Yu Chen, ischenyu.com@outlook.com.
